# A Well-Conserved Archaeal B-Family Polymerase Functions as an Extender in Translesion Synthesis

**DOI:** 10.1128/mbio.02659-21

**Published:** 2022-01-18

**Authors:** Xu Feng, Baochang Zhang, Zhe Gao, Ruyi Xu, Xiaotong Liu, Sonoko Ishino, Mingxia Feng, Yulong Shen, Yoshizumi Ishino, Qunxin She

**Affiliations:** a CRISPR and Archaea Biology Research Center, Microbial Technology Institute and State Key Laboratory of Microbial Technology, Shandong University, Qingdao, China; b Department of Bioscience and Biotechnology, Graduate School of Bioresource and Bioenvironmental Sciences, Kyushu University, Fukuoka, Japan; University of Vienna

**Keywords:** DNA polymerases, Dpo2, translesion DNA synthesis, extender polymerase, mismatch extension, *Sulfolobus*, *Archaea*

## Abstract

B-family DNA polymerases (PolBs) of different groups are widespread in *Archaea*, and different PolBs often coexist in the same organism. Many of these PolB enzymes remain to be investigated. One of the main groups that is poorly characterized is PolB2, whose members occur in many archaea but are predicted to be inactivated forms of DNA polymerase. Here, Sulfolobus islandicus DNA polymerase 2 (Dpo2), a PolB2 enzyme, was expressed in its native host and purified. Characterization of the purified enzyme revealed that the polymerase possesses a robust nucleotide incorporation activity but is devoid of the 3′–5′ exonuclease activity. Enzyme kinetics analyses showed that Dpo2 replicates undamaged DNA templates with high fidelity, which is consistent with its inefficient nucleotide insertion activity opposite different DNA lesions. Strikingly, the polymerase is highly efficient in extending mismatches and mispaired primer termini once a nucleotide is placed opposite a damaged site. This extender polymerase represents a novel type of prokaryotic PolB specialized for DNA damage repair in *Archaea*.

## INTRODUCTION

Cellular organisms code for multiple DNA polymerases that play crucial roles in chromosome duplication and genome integrity maintenance during normal growth and under stressed conditions. Eight different families of DNA polymerases (pols) are known based on their amino acid sequences, and as many as 17 DNA pols are encoded in humans (1). Some polymerases are devoted to chromosome replication (replicase), while others are specialized for DNA damage repair. Bacterial replicases for chromosome replication are of the C-family, and those in the organisms of *Eukarya* and *Archaea* belong to the B-family or D-family (2–4). Replicative polymerases possess both the polymerase and exonuclease domains and replicate undamaged DNA with high fidelity and processivity. In contrast, most specialized DNA pols are of X- and Y-family. These pols are often devoid of any proofreading activity and replicate DNA with reduced fidelity and processivity (1, 5). A noticeable exception of specialized pols is the eukaryotic Pol ζ, a B-family DNA polymerase, which plays important roles in the eukaryotic translesion DNA synthesis (TLS) by functioning as an extender of DNA ends with mismatches and after lesion bypass.

Sulfolobales organisms, such as Sulfolobus islandicus, Sulfolobus acidocaldarius, and *Saccharolobus solfataricus* P2 (formerly Sulfolobus solfataricus), encode four DNA polymerases. These DNA pols were initially named Dpo1, Dpo2, Dpo3, and Dpo4/Dbh (DinB homolog), among which the first three belong to the B family (also known as PolB1, PolB2, and PolB3), whereas the last is a Y-family pol (6–8). DNA pols in S. solfataricus were characterized *in vitro* in different research laboratories, and these analyses have revealed the Dpo1 and Dpo3 enzymes are high-fidelity DNA polymerases exhibiting the 3′–5′ exonuclease activity, and this is consistent with their predicted function in processive DNA replication in this crenarchaeon (9–11). The S. solfataricus Dpo4 represents the most extensively characterized Y-family DNA pol. This enzyme is capable of bypassing various DNA lesions *in vitro* (12–15), suggesting it is responsible for translesion synthesis in this organism. However, the encoding gene does not show any DNA damage-inducible expression in all tested *Sulfolobales* organisms, including S. acidocaldarius, S. solfataricus, and S. islandicus (16–20), and it does not play a role in the targeted mutagenesis, as we have demonstrated with the S. islandicus Δ*dpo4* mutant (21). The only DNA pol gene that does show damage-inducible expression is *dpo2*, coding for a PolB2 enzyme (16–19), and it has been further shown that *dpo2* is solely responsible for the DNA damage-induced mutagenesis in S. islandicus (21). Nevertheless, members of the PolB2 subfamily were regarded as inactive polymerases, since they carry amino acid substitutions at the catalytic center (22). Consequently, whether Dpo2 could be an active polymerase represents a very important question in the TLS study of Dpo2-encoding organisms, and, if so, it would be intriguing to know how this unique DNA pol contributes to DNA damage repair in these archaea.

Here, we biochemically characterized S. islandicus Dpo2. Recombinant Dpo2 protein was obtained from the native host and investigated for its capability of DNA polymerization, proofreading, and lesion bypass. We found that Dpo2 is a robust DNA pol in nucleotide incorporation but is devoid of the 3′–5′ exonuclease activity. This unique DNA pol replicates undamaged DNA with a replication fidelity that is comparable to that of Dpo1, the main replicase of the organism. We further demonstrate that Dpo2 is very inefficient in nucleotide insertion opposite a DNA lesion but is efficient in extending from mispaired ends after nucleotide insertion opposite DNA lesions. Together, these results indicated that the PolB2 enzymes are specialized DNA polymerases that can play very important roles in archaeal DNA damage repair.

## RESULTS

### S. islandicus Dpo2 is an active DNA polymerase devoid of the exonuclease activity.

**FIG 1:**
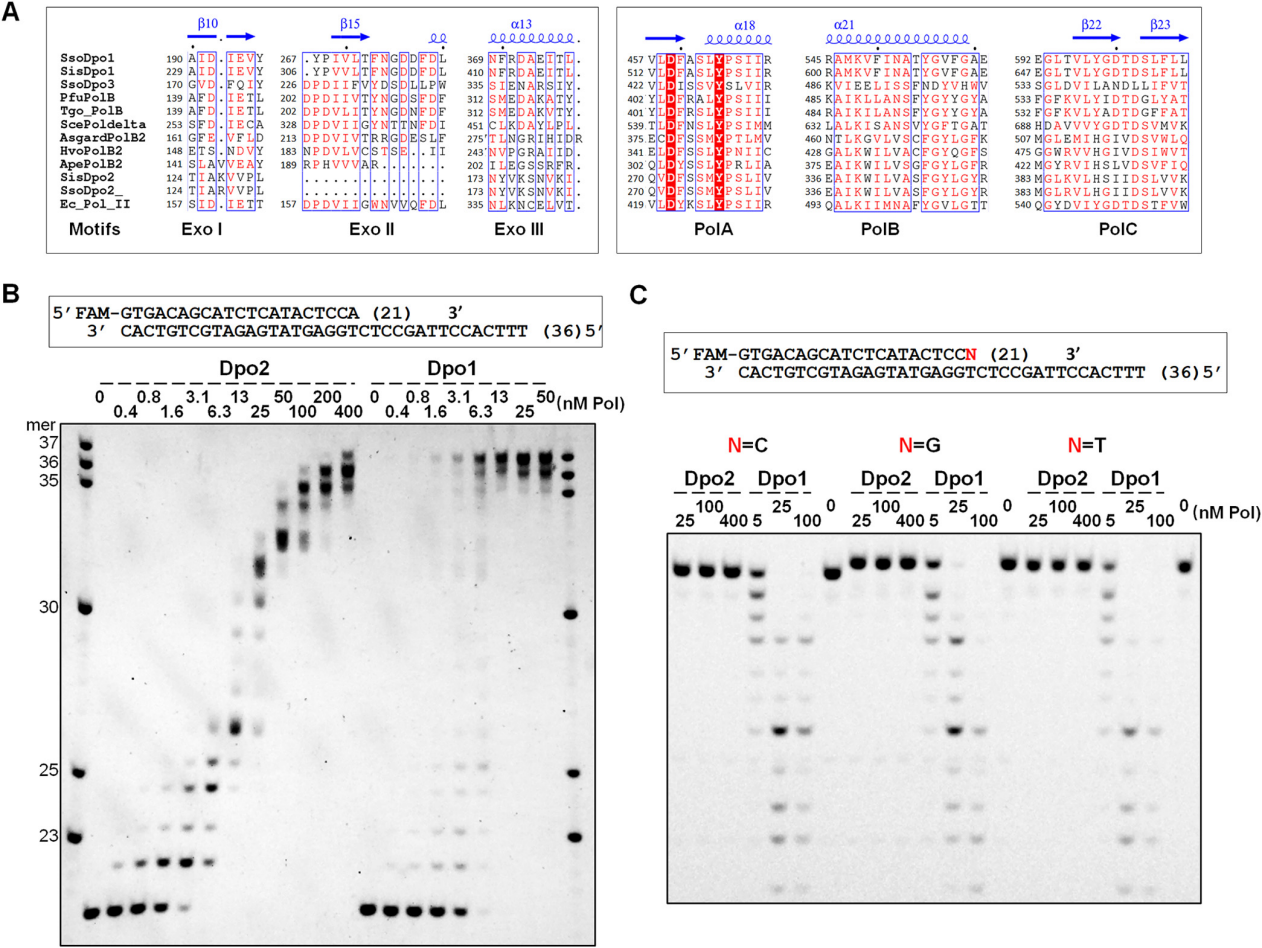
Dpo2 is efficient in primer extension but deficient in proofreading activity. (A) Sequence alignment of a few selected B-family DNA polymerases. Only the selected regions of the exonuclease domain and polymerase domain are shown, and the full sequence alignment is shown in Fig. S1. SsoDpo1, S. solfataricus Dpo1. SisDpo1, S. islandicus Dpo1. SsoDpo3, S. solfataricus Dpo3. PfuPolB, Pyrococcus furiosus PolB. Tgo_PolB, Thermococcus gorgonarius PolB. ScePoldelta, the catalytic subunit of Saccharomyces cerevisiae Pol δ. AsgardPolB2, “*Candidatus* Thorarchaeota archaeon” PolB2. HvoPolB2, Haloferax volcanii PolB2. ApePolB2, Aeropyrum pernix PolB2. Ec_Pol_II, E. coli Pol II. Structures of SsoDpo1 (PDB entry 1S5J) were used as the templates for the structure-based sequence alignment. The secondary structural elements shown above the sequences were retrieved from the structure file of SsoDpo1 (1S5J). (B) Primer extension activities of Dpo2 and Dpo1. Reactions were set up with 50 nM primer template, 100 μM dNTPs, and a concentration gradient of Dpo2 or Dpo1 (indicated above their gel images in each panel). After incubation at 60°C for 10 min, extension products were analyzed by denaturing PAGE. Note that Dpo1 yielded an extension product of 37 nt, which is one nucleotide longer than the template (36 nt), indicative of strong TdT (terminal transferase) activity of the enzyme. In contrast, Dpo2 only showed low TdT activity. (C) Proofreading by Dpo2 and Dpo1. Exonuclease assay was set up with 50 nM mismatched primer template and a gradient concentration of Dpo2 or Dpo1 in the absence of dNTPs. After incubation at 60°C for 5 min, the products were analyzed by denaturing PAGE. N denotes each of the four possible primer terminal nucleotides as indicated.

In a previous work, we showed that *dpo2* is the only DNA polymerase gene essential for DNA damage-induced mutagenesis in S. islandicus (21). This polymerase belongs to the PolB2 subfamily of DNA polymerases that possess a relatively conserved polymerase domain, including conserved PolA, PolB, and PolC motifs that are normally conserved in other B-family DNA polymerases, although its PolC motif carries amino acid substitutions at conserved Y and D (22, 23). Here, we also conducted multiple-sequence alignments with the S. islandicus Dpo2 protein and a selected set of B-family polymerases, including those of Saccharomyces cerevisiae, Escherichia coli, and a few archaea, and their sequence conservation and variations are shown in Fig. 1A and Fig. S1 in the supplemental material. Noticeably, whereas the exonuclease domain is present in the B-family replicative polymerases, it is either absent or much more diverged from the PolB2 enzymes.

10.1128/mBio.02659-21.3 FIG S1 Sequence alignment of a selected set of B-family DNA polymerases. B-family DNA polymerases employed for the analysis include SsoDpo1, S. solfataricus Dpo1; SisDpo1, S. islandicus Dpo1; SsoDpo3, S. solfataricus Dpo3; PfuPolB, Pyrococcus furiosus PolB; Tgo_PolB, Thermococcus gorgonarius PolB; ScePoldelta, the catalytic subunit of Saccharomyces cerevisiae Pol δ; AsgardPolB2, “*Candidatus* Thorarchaeota archaeon” PolB2; HvoPolB2, Haloferax volcanii PolB2; ApePolB2, Aeropyrum pernix PolB2; and Ec_Pol_II, E. coli Pol II. Structures of SsoDpo1 (1S5J) were used as the templates for the structure-based sequence alignment. The secondary structural elements shown above the sequences were retrieved from the structure file of SsoDpo1 (1S5J). Download FIG S1, TIF file, 2.2 MB. Copyright © 2022 Feng et al. 2022 Feng et al. https://creativecommons.org/licenses/by/4.0/ This content is distributed under the terms of the Creative Commons Attribution 4.0 International license.

To experimentally characterize PolB2, we attempted to express the S. islandicus Dpo2 in E. coli cells. However, the recombinant protein formed almost exclusively aggregates in inclusion bodies, indicative of improper folding of Dpo2 in the mesophilic host. We then chose to express the Dpo2 protein in its native host and purified the native form of protein into an apparent homogeneity (Fig. S2). The purified Dpo2 was then assayed for the basic properties and for the optima of the primer extension reaction (Fig. S3), using the substrate shown in Fig. 1B. These included determination of its optimal values in reaction pH, temperature, and salt content as well as the metal ion preference. As shown in Fig. S3A, the activity of Dpo2 increased along with the increase of pH from 6.0 to 8.0 before shallowing down at pH 8.8. The longest synthesized DNA fragments appeared in the reactions of pH 8.0 and 8.4, suggesting this pH range is optimal for the polymerase. As a result, subsequent optimization of the Dpo2 assay was conducted with buffers containing 50 mM Tris-Cl, pH 8.0. The effect of salt on the activity of Dpo2 was tested with both KCl and NaCl. We found that the S. islandicus Dpo2 was most active with the low-salt buffer, and, in fact, 80 mM KCl or 20 mM NaCl could already inhibit the Dpo2 activity (Fig. S3B). Six different divalent metal ions (Mg^2+^, Mn^2+^, Ca^2+^, Zn^2+^, Ni^2+^, and Fe^2+^) were tested for their capability of supporting the polymerization activity, and this revealed that both magnesium and manganese ions supported the Dpo2 activity. Furthermore, Dpo2 showed higher activity in the presence of equal concentration of the manganese ion relative to the magnesium ion (Fig. S3D), as reported for many specialized DNA polymerases (24–28). Nevertheless, Mg^2+^ was used in the following analysis, considering a much higher physiological concentration for this metal ion in different cells. In addition, the optimal reaction temperature and deoxynucleotide triphosphate (dNTP) concentration determined for Dpo2 were 55 to 65°C and 100 to 500 μM, respectively (Fig. S3). To this end, the optimized buffer system for Dpo2 was defined as 50 mM Tris-HCl, pH 8.0, 40 mM KCl, 0.1 mg/ml bovine serum albumin (BSA), 10 mM MgCl_2_, 100 μM dNTPs, which was employed for all subsequent assays with the reactions carried out at 60°C.

10.1128/mBio.02659-21.4 FIG S2 SDS-PAGE analysis of purified Dpo2 and Dpo1 proteins from the native host. (A) Dpo2 with a theoretical size of 64,927 Da. (B) Dpo1 with a theoretical size of 101,205 Da. Mass spectrometry confirmed that band 1, corresponding to a size of 100,000, was Dpo1, as were two smaller species of ∼60,000 (2) and ∼40,000 (3), which presumably resulted from Dpo1 degradation. The smallest species (4) were identified as a mixture of Dpo1 degradation and PBP1 (SiRe_1861), which is probably part of the Dpo1 holoenzyme, as has been shown for SsoDpo1 (9). Download FIG S2, TIF file, 0.1 MB. Copyright © 2022 Feng et al. 2022 Feng et al. https://creativecommons.org/licenses/by/4.0/ This content is distributed under the terms of the Creative Commons Attribution 4.0 International license.

10.1128/mBio.02659-21.5 FIG S3 Optimization of the reaction condition. DNA substrates were the same as those shown in Fig. 1. Reaction mixtures were set up with 37.5 nM Dpo2 and 50 nM substrates. (A) Optimization of the reaction pH at 60°C. N, no enzyme control. (B) Optimization of salt concentration at 60°C using the Tris-HCl buffer, pH 8.0. (C) Optimization of dNTP concentration, using Tris-HCl, pH 8.0, buffer containing 40 mM KCl and 0.1 mg/ml BSA at 60°C. (D) Metal ion dependence of Dpo2. The reactions were carried out with Tris-HCl, pH 8.0, containing 40 mM KCl and 0.1 mg/ml BSA at 60°C. Mg^2+^ was used, since some of the following analyses involved the use of multiple DNA Pol enzymes and a much higher physiological concentration of Mg^2+^ has been reported than for other metal ions. (E) Determination of the optimal reaction temperature using Tris-HCl, pH 8.0, buffer containing 40 mM KCl and 0.1 mg/ml BSA at the indicated temperature. (F) Optimization of Mg^2+^ concentration. The optimized buffer system for Dpo2 contains 50 mM Tris-HCl, pH 8.0, 40 mM KCl, 0.1 mg/ml BSA, 10 mM MgCl2, 100 μM dNTPs. In the single-nucleotide incorporation assay and kinetics analysis, the dNTPs mixtures were omitted and a single dNTP with indicated concentration was instead used. Download FIG S3, TIF file, 0.4 MB. Copyright © 2022 Feng et al. 2022 Feng et al. https://creativecommons.org/licenses/by/4.0/ This content is distributed under the terms of the Creative Commons Attribution 4.0 International license.

Using the optimized reaction system, we examined the primer extension activity of Dpo2 compared with Dpo1, the replicase of this crenarchaeon (9). The enzyme concentrations tested for Dpo2 and Dpo1 were 0.4 to 400 nM and 0.4 to 50 nM, respectively, and this revealed that Dpo2 manifested DNA polymerization at a concentration as low as 3 nM, and the amount of primer consumed by Dpo2 in this assay was comparable to that converted by Dpo1 at the identical or a very similar enzyme concentration (Fig. 1B). These results indicated that Dpo2 exhibits robust nucleotide incorporation activity. Noticeably, while the replicase readily extended the primer into full-length products (with 13 nM Dpo1), the Dpo2 polymerization yielded DNA fragments of different sizes in the reaction mixtures with the same or a higher enzyme concentration (Fig. 1B). These data suggested that S. islandicus Dpo2 is a distributive polymerase relative to the processive Dpo1 enzyme.

To test if S. islandicus Dpo2 could perform proofreading during DNA synthesis, the mismatched primer templates (including T:C, T:G, and T:T mismatches) were mixed individually with Dpo2 (25, 100, or 400 nM) as well as Dpo1 (5, 25, or 100 nM), the latter of which is known to possess the 3′–5′ exonuclease activity. After incubation at 60°C for 5 min, samples were analyzed by denaturing PAGE. As shown in Fig. 1C, while 25 nM Dpo1 effectively degraded all three primers from the 3′ terminus, generating a ladder of degraded oligonucleotides, 16-fold more Dpo2 enzyme did not show any detectable 3′-5′ exonuclease activity, since full-length primers remained intact in the reaction mixture with 400 nM Dpo2.

Taken together, S. islandicus Dpo2 represents a unique PolB exhibiting robust nucleotide incorporation, poor processivity, and no detectable exonuclease activity.

### Dpo2 replicates undamaged DNA with high fidelity.

Next, we sought to decipher kinetic parameters of nucleotide incorporation by this unique B-family enzyme, using the steady-state kinetic assay described in Materials and Methods. Dpo2 was evaluated for the fidelity of nucleotide incorporation opposite each of the four template bases. As summarized in Table 1, Dpo2 clearly can discriminate correct and incorrect incoming nucleotide, as it incorporated correct nucleotides opposite different template bases with the highest efficiency (*k*_cat_/*K_m_*) and with the lowest *K_m_* values. Overall, insertion of a wrong nucleotide (misincorporation) by Dpo2 occurred at a frequency ranging from 2.8 × 10^−5^ (for inserting a C opposite a template base C) to 5.37 × 10^−4^ (for inserting a G opposite an A). Thus, the misincorporation frequency of this unique PolB on four different template bases is from 10^−4^ to 10^−5^, falling into the same range of the replication fidelity by the S. solfataricus replicase Dpo1 at 37°C (29) and its exonuclease-minus mutant, Dpo1 exo^−^, at 55°C (10). Thus, Dpo2 is a high-fidelity DNA polymerase on undamaged DNA templates.

**TABLE 1:** Steady-state kinetic parameters of deoxynucleotide incorporation by Dpo2 on undamaged DNA^*a*^

Template base	Incoming dNTP	*K_m_* (μM)	*k*_cat_ (min^−1^)	*k*_cat_/*K_m_* (μM^−1^ min^−1^)	*f_i_*_nc_
A	A	1,205 ± 201	0.0112 ± 0.0022	9.3 × 10^−6^	1.91 × 10^−4^
	T	39.9 ± 14.9	1.94 ± 0.41	4.9 × 10^−2^	1
	G	902 ± 188	0.0235 ± 0.00076	2.6 × 10^−5^	5.37 × 10^−4^
	C	488 ± 56.2	0.00587 ± 0.00015	1.2 × 10^−5^	2.48 × 10^−4^
T	A	118 ± 10.6	11.7 ± 5.91	9.9 × 10^−2^	1
	T	1,616 ± 91	0.0234 ± 0.0021	1.5 × 10^−5^	1.46 × 10^−4^
	G	1,454 ± 131	0.0299 ± 0.0040	2.1 × 10^−5^	2.08 × 10^−4^
	C	413 ± 83.6	0.00156 ± 0.00028	3.8 × 10^−6^	3.81 × 10^−5^
G	A	982 ± 75.9	0.0118 ± 0.0020	1.2 × 10^−5^	1.17 × 10^−4^
	T	1,773 ± 107	0.0279 ± 0.0077	1.6 × 10^−5^	1.53 × 10^−4^
	G	2,474 ± 628	0.0257 ± 0.0053	1.0 × 10^−5^	1.01 × 10^−4^
	C	40.6 ± 5.78	4.17 ± 0.92	1.0 × 10^−1^	1
C	A	1,230 ± 168	0.0241 ± 0.0047	1.9 × 10^−5^	3.17 × 10^−4^
	T	1,428 ± 130	0.0084 ± 0.0015	5.9 × 10^−6^	1.00 × 10^−4^
	G	62.1 ± 8.54	3.63 ± 0.29	5.8 × 10^−2^	1
	C	684 ± 231	0.00112 ± 0.00032	1.6 × 10^−6^	2.80 × 10^−5^

a *K_m_* and *k*_cat_ values were determined by quantification of gel bands corresponding to substrates and products using ImageQuantTL, and the data were fitted into the Michaelis-Menten equation using GraphPad Prism. The nucleotide misincorporation ratio (*f*_inc_) was expressed as (*k*_cat_/*K_m_*)_incorrect_/(*k*_cat_/*K_m_*)_correct_. SD values are standard deviations from three independent experiments.

### Dpo2 is proficient in extension of mismatched primer termini.

To test if Dpo2 could extend mismatched base pair ends, we determined the ability of Dpo2 to elongate 4 matched and 12 mismatched primer templates (Table S2) using the steady-state kinetics assay. As summarized in Table 2, the frequencies of Dpo2 (ƒ^0^_ext_) in mismatch extension from A:A, A:G, and G:T mispairs were estimated to 4.5 × 10^−1^, 1.3 × 10^−1^, and 2.6 × 10^−1^, respectively, and ƒ^0^_ext_ values for extension from most of the rest of the mispairs were found to be on the order of 10^−2^. These results indicated that Dpo2 can effectively extend mismatched primer termini.

**TABLE 2:** Steady-state kinetics parameters for mispair extension by Dpo2^*a*^

Template base	Primer base	*K_m_* (μM)	*k*_cat_ (min^−1^)	*k*_cat_/*K_m_* (μM^−1^ min^−1^)	ƒ^0^_ext_
A	A	55.0 ± 5.0	1.76 ± 0.067	3.2 × 10^−2^	4.5 × 10^−1^
	T	55.2 ± 14.5	3.96 ± 0.34	7.2 × 10^−2^	1
	G	323 ± 84.7	3.09 ± 0.21	9.6 × 10^−3^	1.3 × 10^−1^
	C	1,129 ± 222	1.03 ± 0.095	9.1 × 10^−4^	1.3 × 10^−2^
T	A	19.1 ± 2.66	35.9 ± 3	1.9	1
	T	325 ± 59.6	1.43 ± 0.34	4.4 × 10^−3^	2.3 × 10^−3^
	G	187 ± 22	21.5 ± 8.91	1.1 × 10^−1^	6.1 × 10^−2^
	C	886 ± 76.1	19.7 ± 5.89	2.2 × 10^−2^	1.2 × 10^−2^
G	A	514 ± 57.2	0.271 ± 0.045	5.3 × 10^−4^	3.2 × 10^−2^
	T	476 ± 112	2.01 ± 0.44	4.2 × 10^−3^	2.6 × 10^−1^
	G	517 ± 123	0.125 ± 0.027	2.4 × 10^−4^	1.5 × 10^−2^
	C	86.1 ± 11	1.41 ± 0.13	1.6 × 10^−2^	1
C	A	405 ± 33.6	1.11 ± 0.042	2.8 × 10^−3^	1.7 × 10^−2^
	T	479 ± 80	0.662 ± 0.045	1.4 × 10^−3^	8.7 × 10^−3^
	G	21.6 ± 6.3	3.42 ± 0.35	1.6 × 10^−1^	1
	C	233 ± 54.2	0.461 ± 0.05	2.0 × 10^−3^	1.2 × 10^−2^

a Extension efficiency was examined with dGTP, the next correct nucleotide.

10.1128/mBio.02659-21.2 TABLE S2 DNA substrates used in this study. Download Table S2, DOCX file, 0.04 MB. Copyright © 2022 Feng et al. 2022 Feng et al. https://creativecommons.org/licenses/by/4.0/ This content is distributed under the terms of the Creative Commons Attribution 4.0 International license.

**FIG 2:**
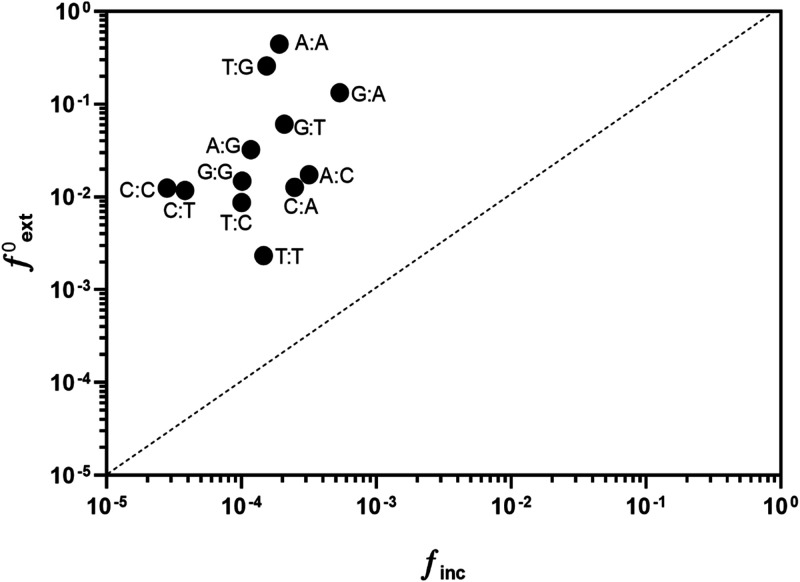
Dpo2 is proficient in extension of mismatched primer termini. Values of *ƒ*^0^_ext_ (the ratio of the apparent *k*_cat_/*K_m_* of extension from the mismatched base pair to the apparent *k*_cat_/*K_m_* of extension from matched base pair) presented in Table S4 were plotted against the values of misincorporation frequency (*ƒ*_inc_) shown in Table S3. The dashed line corresponds to *ƒ*^0^_ext_ = *ƒ*_inc_.

To better illustrate the properties of Dpo2 in nucleotide polymerization, its ƒ_inc_ values (*x* axis) for inserting a wrong nucleotide opposite a template (Table 1) were plotted against the corresponding ƒ^0^_ext_ values (*y* axis) extending from that mispair (Table 2). As shown in Fig. 2, data points are scattered at the upper left. These data indicated that Dpo2 exhibits a much higher efficiency in the mismatch extension than in the mispair formation (10^−1^ to 10^−3^ versus 10^−4^ to 10^−5^), and the enzyme preferably extends primer termini ending with dG, dA, and wobble base pairs (T:G and G:T). These results are in strict contrast to an almost equal efficiency in mispair formation and in mismatch extension for Dpo4 and other nonextender DNA polymerases (46, 59), whose data points scatter along the dashed line in Fig. S3. Thus, we reasoned that Dpo2 could function as a mismatch extender in *Sulfolobus*.

### Dpo2 efficiently extends primer termini opposite the lesion site.

**FIG 3:**
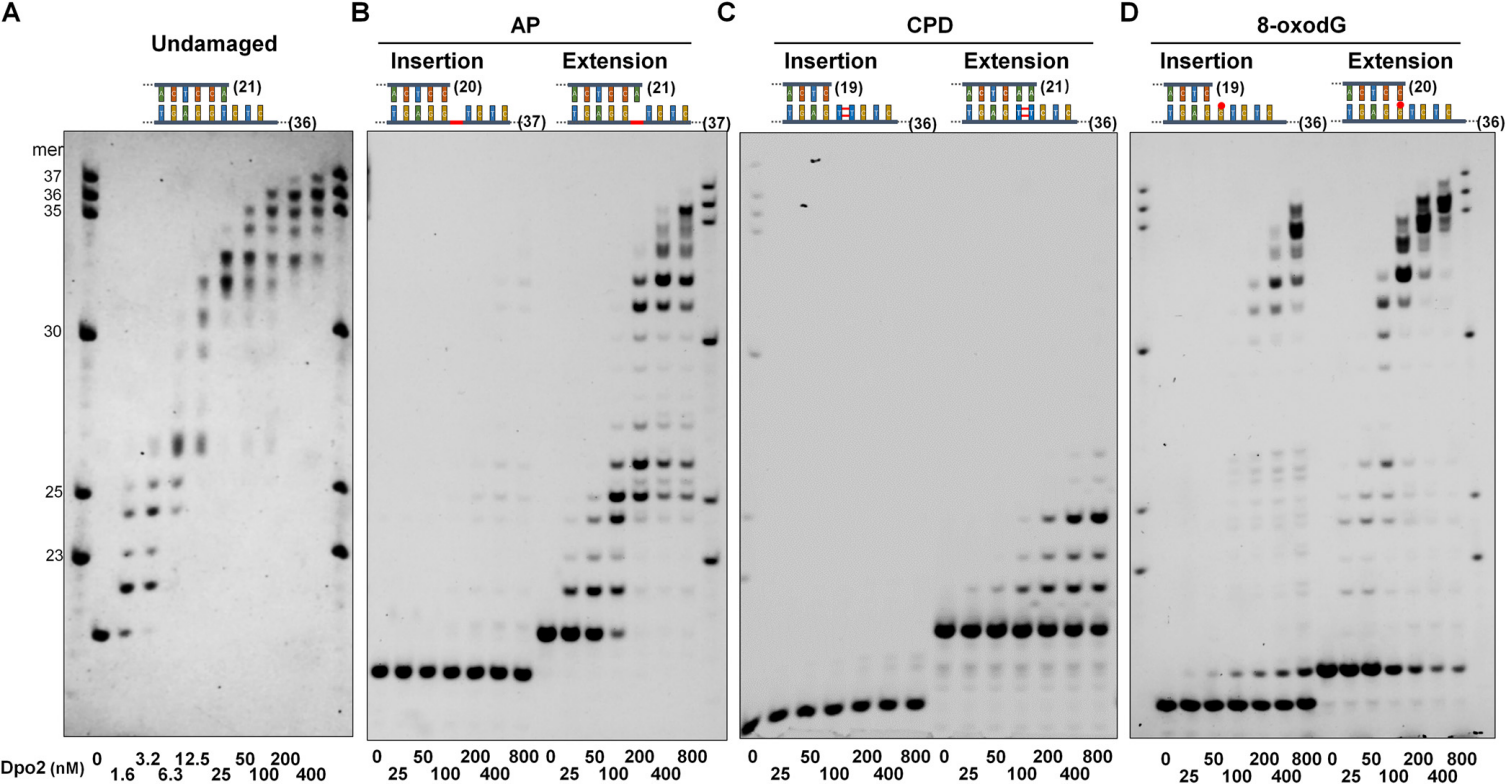
Dpo2 efficiently extends primer termini opposite the lesion site. DNA substrates employed for primer extension assay are illustrated above the corresponding gel images. Templates in the substrates are of 4 different types. (A) Undamaged template, which is lesion-free (undamaged); (B) template carrying an AP lesion, which is highlighted in red in the backbone; (C) template carrying TT-CPD, which is shown as two parallel bars adjoined with two red lines; (D) template containing 8-oxodG (shown as a “G” base carrying a red hat). Numbers in parentheses indicate lengths of primers and templates in each substrate. Primer extension was conducted with reaction mixes containing Dpo2 of varied concentrations (indicated below gel images) and analyzed by denaturing PAGE. Numbers in the size marker denote the lengths of nucleotides.

The exceptional capability of mispair extension by Dpo2 prompted us to test its activity in translesion DNA synthesis. Three DNA lesions were chosen for the experiment, including the AP site, cis-syn cyclobutane pyrimidine dimer (CPD), and 8-oxo-7,8-dihydro-2′-deoxyguanosine (8-oxodG), all of which are common forms of DNA damage encountered by a thermophilic acidophile. The DNA template designed for the AP site bypass experiments was a 37-nucleotide (nt) oligonucleotide containing a synthetic abasic site (tetrahydrofuran analogue) at the 17th position (Table S2). Two primers were then designed, one that extends to the −1 position of the AP site of the template (for the TLS insertion assay) and the other to the position opposite the abasic site (for the TLS extension assay). Annealing of the DNA template with each of the primers yielded two series of primer-template substrates for the experiments of TLS insertion and TLS extension, respectively. Seven reactions were set up for each assay, in which only the Dpo2 content varied within the indicated range. After incubation for 10 min, primer extension products were analyzed by denaturing polyacrylamide gel electrophoresis (PAGE). As shown in Fig. 3B, in the AP insertion assay, the signal of primer extension products was hardly detectable at the position across the lesion and beyond even in the presence of 800 nM Dpo2. This enzyme concentration is 100-fold higher than the efficient primer extension on an undamaged DNA template by the polymerase (Fig. 3A). Intriguingly, when the abasic site was covered by the terminal nucleotide of a primer (TLS extension), +1 extension product was already detected with 25 nM Dpo2, the lowest enzyme concentration tested here. Furthermore, the size of extension products increased along with the elevation of the Dpo2 concentration, and all primers were converted into longer products at 200 nM enzyme (Fig. 3A). These data indicated that Dpo2 works as an extender polymerase in the TLS bypass of abasic sites.

When a DNA template carrying a CPD was employed, we found again that Dpo2 failed to insert any nucleotide opposite the lesion, but it was capable of extending mispaired primer ends, albeit at an efficiency lower than that of the extension of the AP-contained mispaired primer ends (Fig. 3C). In the case of the 8-oxodG bypass reaction, Dpo2 incorporated a nucleotide across the 8-oxodG lesion for 53.8% of the template even at the highest concentration (800 nM) of the enzyme tested in this study, indicative of very weak activity in the TLS insertion. In contrast, the Dpo2 extension is robust, since a comparable amount of substrate has rapidly been extended at an 8-fold lower concentration (100 nM) (Fig. 3D).

### S. solfataricus Dpo2 is also a robust DNA polymerase lacking proofreading.

**FIG 4:**
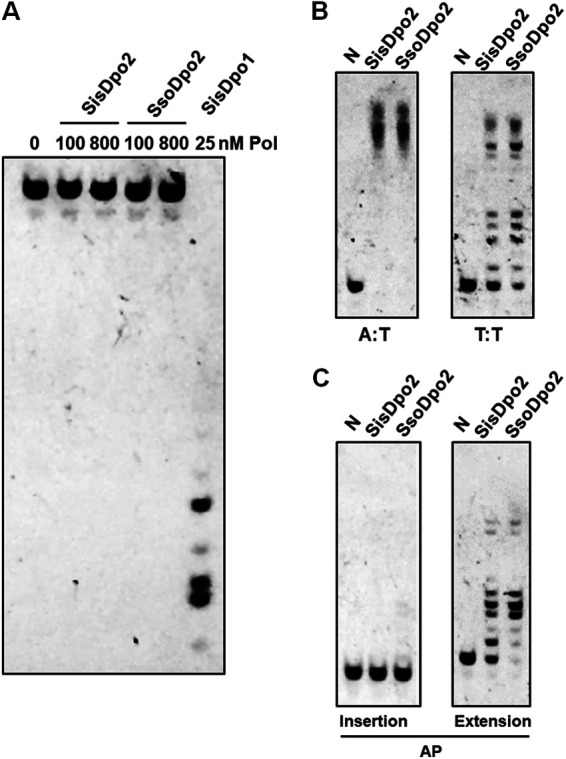
*Sso*Dpo2 has activity similar to that of *Sis*Dpo2. (A) Exonuclease assay. The assay was set up with 50 nM substrate and enzyme concentrations indicated above each lane. Reactions were conducted at 60°C for 5 min. (B) Extension of the undamaged substrate (A:T) and mismatched substrate (T:T). Substrates used for the assays are the same as those shown in Fig. 1. Each reaction mix contains 100 nM DNA polymerase and 50 nM substrate. N, no enzyme control. (C) TLS insertion and extension. Assays were set up with the substrates shown in Fig. 3B. Primer extension reactions were conducted with 50 nM substrates.

We noticed that our results with S. islandicus Dpo2 are in contrast to those obtained with the S. solfataricus Dpo2 (*Sso*Dpo2) that was expressed in an E. coli host in a previous work. In the latter, only weak activities were observed in polymerization and in proofreading for the heterologously expressed form of *Sso*Dpo2 (29). Since Dpo2 proteins of S. solfataricus and S. islandicus share 91% and 96% sequence identity and similarity, it is very unlikely the two proteins would exhibit any major differences in enzymatic properties. *Sso*Dpo2 was then expressed in S. islandicus, and the enzyme was purified and characterized along with S. islandicus Dpo2 (Fig. S5). We found that the recombinant *Sso*Dpo2 obtained from the *Sulfolobus* host does not possess any detectable 3′–5′ exonuclease activity either, which is consistent with the lack of Exo motifs. The PolB enzyme is highly efficient in nucleotide incorporation, exhibiting a strong propensity in mismatch extension and TLS extension during DNA synthesis (Fig. 4).

10.1128/mBio.02659-21.6 FIG S4 Comparison of mismatch extension ability of extender and nonextender DNA polymerases. The ƒinc and ƒ0ext values of *Saccharolobus solfataricus* Dpo4 (SsoDpo4), Saccharomyces cerevisiae Pol ζ (ScePol ζ), and S. cerevisiae Pol η (ScePol η) were retrieved from references 46, 56, and 57, respectively. Download FIG S4, TIF file, 0.4 MB. Copyright © 2022 Feng et al. 2022 Feng et al. https://creativecommons.org/licenses/by/4.0/ This content is distributed under the terms of the Creative Commons Attribution 4.0 International license.

10.1128/mBio.02659-21.7 FIG S5 Optimal temperature of SsoDpo2. (A) SDS-PAGE analysis of purified SisDpo2 and SsoDpo2 proteins. SisDpo2, Sulfolobus islandicus Dpo2. SsoDpo2, S. solfataricus Dpo2. Both proteins were expressed in the S. islandicus host. (B) The optimal temperature of SsoDpo2. The reaction mixture contained 17.5 nM SsoDpo2 protein and 50 nM substrate, used in Fig. 1B, in the presence of 100 μM dNTPs. The experiment was carried out at the temperature indicated above each lane for 5 min. Download FIG S5, TIF file, 0.2 MB. Copyright © 2022 Feng et al. 2022 Feng et al. https://creativecommons.org/licenses/by/4.0/ This content is distributed under the terms of the Creative Commons Attribution 4.0 International license.

Taken together, the above-described results indicated that PolB2s are very inefficient in misincorporation and TLS insertion but show robust activity in mismatch and extension steps of lesion bypass. Thus, they probably function as an extender DNA polymerase in translesion DNA synthesis.

## DISCUSSION

Members of PolB2 enzymes are widespread in *Archaea*. This group of DNA polymerases is unique, since its members carry deletions or radical variation in the Exo motifs and variations in key amino acid residues of the PolC motif that are very conserved in all other groups of PolB enzymes (22, 23). For this reason, PolB2 members were once regarded as a group of inactivated DNA polymerases. Here, we report that both S. islandicus and S. solfataricus Dpo2 enzymes are efficient in nucleotide incorporation, since their primer extension activities are comparable to that of Dpo1, the replicase that coexists in these archaeal organisms. We further show that the archaeal PolB2s lack any detectable 3′–5′ exonuclease activity, and they are proficient in mismatch extension and extending primer termini opposite DNA lesions. Our results suggest the archaeal PolB2 enzymes represent a novel type of PolB that play important roles in translesion DNA synthesis. In addition, a clade of bacterial PolB2 (G2) has been found to be evolutionarily related to archaeal PolB2 (2, 23). Since these bacterial orthologues share the same mutations in ExoII and PolC with the archaeal PolB2 enzymes (23), they may also function as an extender polymerase, as demonstrated for the archaeal PolB2.

Our identification of the robust DNA polymerase activity for *Sulfolobus* Dpo2 has yielded important insights into the mechanisms of DNA synthesis. B-family DNA polymerases share conserved motifs, three of which (Exo I, II, and III) are located in the proofreading domain, while the remaining (e.g., PolA, -B, and -C) are in the polymerase domain (Fig. 1A). The PolB2 group of DNA pols exhibits numerous variations in these motifs. The *Sulfolobus* Dpo2 proteins investigated in this work represent the smallest PolB2 known to date (see Fig. S6 in the supplemental material). These enzymes lack most of the conserved amino acids in the proofreading domain and exhibit large variations in the PolC motif. The latter is in contrast to the members of other PolB groups, since their PolCs have the YxDTD invariant motif, which is mutated into HxxxD in PolB2. It has been reported that the PolC motif of B-family DNA pols plays a key role in primer/template recognition and participates in the coordination of the catalytic Mg^2+^ that is essential for the polymerization reaction (30, 31). Nevertheless, previous works have already shown that the two Asp residues in PolC are not equally important for catalysis of DNA polymerization. Structural interrogation of a few B-family DNA polymerases has revealed that the second aspartate is responsible for the metal ion coordination, and the first Asp is oriented away from the activity center (31–35). Since mutagenesis of the first Asp greatly reduces the activity of the human Pol α and two viral replicases (36–38), this acidic amino acid, although not directly involved in catalysis, still plays an important role in polymerase activity. However, our work shows that *Sulfolobus* Dpo2 enzymes, although lacking the first Asp of PolC, are as active as the Dpo1 replicase in nucleotide incorporation. This suggests the first Asp in the PolC motif is functionally replaced by His, an invariant amino acid in the PolC motif of the PolB2 enzymes (Fig. 1A). Alternatively, the functional complementation may also be accomplished by the Asp in the invariable D(K/R) motif located in a flexible loop near the polymerase active site, as suggested in a previous work (23). In addition, mutation of amino acid residues adjacent to the catalytic Asp in the PolC motif of E. coli Pol I or Thermus aquaticus (Taq) polymerase impairs their mismatch extension ability (39, 40). To this end, we reason that the sequence variation at the PolC motif in the PolB2 enzymes reflects their adaptation to their specialized function in DNA repair, which apparently requires robust polymerase activity and the tolerance of DNA damage, whereas their replication processivity and proofreading are disfavored.

10.1128/mBio.02659-21.8 FIG S6 Phylogeny of PolB2 proteins. The phylogenetic tree was constructed using sequences of PolB2s extracted from NCBI, and the analysis was performed on the Phylogeny.fr platform (58). The size of each PolB2 was indicated by the numbers in parentheses (aa). Download FIG S6, TIF file, 1.6 MB. Copyright © 2022 Feng et al. 2022 Feng et al. https://creativecommons.org/licenses/by/4.0/ This content is distributed under the terms of the Creative Commons Attribution 4.0 International license.

It is notable that the properties of the recombinant SsoDpo2 we have obtained from S. islandicus, a homologous host, are very different from the same enzyme yielded from heterologous expression in E. coli (29). While the E. coli recombinant SsoDpo2 (500 nM) exhibits optimal activity at 50°C and the activity is greatly reduced at 60°C and completely inactivated at 70°C (29), the optimal temperature for the *Sulfolobus*-expressed SsoDpo2 (17.5 nm) is 50 to 65°C, and the enzyme is still very active at 80 to 90°C (Fig. S5). We reason that the observed differences can be attributed to differences in posttranslational modifications (PTMs) present in proteins produced in the thermophilic host versus those synthesized in the mesophilic host (41). Indeed, in a comparative study of a recombinant S. islandicus esterase produced in S. islandicus versus that produced in E. coli, the homologously expressed protein is much more active than the heterologously expressed version of the same enzyme (42). In addition, Dpo2 contains 7 cysteine residues, and their potential for generating intra- and/or intermolecular disulfide bonds may differ strongly in a different genetic background, which also contributes to the differences observed between the two forms of SsoDpo2 recombinant protein.

Our characterization of the archaeal Dpo2 enzymes has revealed that PolB2 enzymes exhibit several distinctive biochemical features, including (i) the lack of a proofreading activity, (ii) that its promiscuous extension of mispaired primer ends can fix mismatches, and (iii) that its capacity in primer extension around the lesion site may generate mutations. The unique features are consistent with their possible functions in DNA damage repair in these crenarchaea, as recently revealed from our genetic analyses in S. islandicus using the gene disruptant strains for *dpo2*, *dpo3*, and *dpo4*. Comparison of their phenotypes with that of the wild-type reference has revealed that Dpo2 is solely responsible for the targeted mutagenesis in this crenarchaeon (21). These unique features of Dpo2 may have provided the molecular mechanisms for the generation of the Dpo2-dependent targeted mutagenesis observed in our genetic study (21). In this regard, Dpo2 is analogous to the eukaryotic Pol ζ, since its deficiency also reduces the targeted mutations in yeast (43, 44), and this B-family DNA polymerase is also known for the lack of proofreading activity and exceptional ability in mismatch extension (45). Considering Pol ζ works in concert with Y-family DNA polymerases (Pol ι or Pol η) in a two-polymerase mechanism for AP lesion bypass in *Eukarya* (46, 47), the identification of PolB2 enzymes as an extender polymerase raises an intriguing question about whether this unique DNA polymerase can act in concert with other DNA polymerases to facilitate lesion bypass in the domain of *Archaea*.

## MATERIALS AND METHODS

### *Sulfolobus* strains and growth conditions.

S. islandicus E233S (Δ*pyrEF* Δ*lacS*) (48), derived from S. islandicus REY15A, the wild-type strain (49), was employed as the host for expression of recombinant DNA polymerases, including S. islandicus Dpo2 and Dpo1 and S. solfataricus Dpo2. *Sulfolobus* strains were grown in SCV (0.2% sucrose, 0.2% Casamino Acids, 1% vitamin solution plus basic salts) or ACV (0.2% d-arabinose, 0.2% Casamino Acids, 1% vitamin solution plus basic salts) medium at 78°C as previously described (50).

### Expression and purification of DNA polymerases from S. islandicus.

The S. islandicus Dpo2 expression plasmid was constructed previously (21). Dpo1 (SiRe_1451)- and SsoDpo2 (Sso1459)-encoding genes were amplified by PCR using corresponding oligonucleotides listed in Table S1 in the supplemental material and individually cloned into pSeSD, an arabinose-inducible expression vector (50). Construction of the expression plasmids, expression of the DNA pol genes, and purification of the encoded proteins from S. islandicus E233S were conducted as previously described (51). Briefly, 20 to 500 ng plasmid DNA was used for electroporation transformation for each plasmid, the colonies appearing on the selective plates (SCV) were checked for DNA insert by colony PCR, and the target genes were verified by sequencing of the PCR product. Transformants carrying each expression plasmid were first cultured in SCV, the noninduction medium, for cell growth. The cultures then were transferred into ACV, the induction medium, for protein expression. Cell mass was harvested from ca. 11 liters of ACV cultures and used for the purification of Dpo2 and Dpo1 individually by the following procedures. Cell pellets were resuspended in buffer A (50 mM Tris-HCl, 200 mM NaCl, 30 mM imidazole, pH 7.5) supplemented with 1× protease inhibitor cocktails and 10 μg/ml DNase I. Cell lysates were obtained by passing the cell suspension through a high-pressure homogenizer (JNBIO). Cell debris in the lysates was removed by centrifugation at 15,000 × *g* for 40 min, and the supernatant was filtered through a 0.45-μm filter. The clarified supernatant was then applied to a Histrap HP column (Cytiva) and the target protein bound to the Ni column via the specific His tag-Ni ion interaction, which was then eluted with buffer B (50 mM Tris-HCl, 200 mM NaCl, 500 mM imidazole, pH 7.5). Further purification of recombinant protein was different for Dpo2 and Dpo1. In the case of Dpo2, pooled elution fractions were diluted using buffer C (20 mM Tris-HCl, pH 8.0) and applied onto a heparin HP column (Cytiva). Proteins bound to the heparin column were then eluted by a linear gradient of buffer D (20 mM Tris-HCl, 1 M NaCl, pH 8.0) over a 25× column volume. Further purification of Dpo1 was conducted by size exclusion chromatography with a Superdex 200 increase 10/300 GL column (Cytiva). Fractions containing each DNA pol of high purity were pooled and concentrated using a 10K protein concentrator (Millipore). Concentrated proteins were preserved at −20°C in the presence of 50% glycerol. The concentration of each protein was determined using a Bradford assay (52), with BSA at known concentrations as standards.

10.1128/mBio.02659-21.1 TABLE S1 Oligonucleotides used in this study. Download Table S1, DOCX file, 0.03 MB. Copyright © 2022 Feng et al. 2022 Feng et al. https://creativecommons.org/licenses/by/4.0/ This content is distributed under the terms of the Creative Commons Attribution 4.0 International license.

### DNA substrates.

All synthetic oligonucleotides, including unlabeled, 6-carboxyfluorescein (FAM)-labeled primers, undamaged templates, and templates containing base modifications, were synthesized and purified by high-performance liquid chromatography (HPLC) at Genewiz (Suzhou, CN) or Sangon Biotech (Shanghai, CN). The exception was a CPD-containing oligonucleotide, which was synthesized and purified by Gene Link (Elmsford, NY, USA). The sequence of primers and templates are listed in Table S1. DNA substrates were prepared by annealing corresponding primer stand and template strand at a 1:1.5 ratio using a thermal cycler in which the temperature was decreased by 0.2°C each cycle for 350 cycles after denaturation at 95°C for 5 min.

### Optimization of reaction conditions.

The optimal pH was determined using a Bis-Tris-based buffer system in the pH range of 6.0 to 7.2 and a Tris-HCl system in the range of 6.8 to 8.8. Salt concentrations of NaCl and KCl were screened in Tris-HCl buffer, pH 8.0. At optimal pH and salt concentration, the reaction temperature and concentration of metal ions was optimized. From these experiments, the optimal condition was determined for the Dpo2 reaction in the solution containing 50 mM Tris-HCl (pH 8.0), 40 mM KCl, 10 mM MgCl_2_, 0.1 mg/ml BSA at 60°C, and it was used for the following analyses.

### Primer extension assay.

The primer extension reaction was set up in a 10-μl reaction system containing 50 nM substrates, DNA polymerases at the indicated concentrations, 100 μM either all four dNTPs or each dNTP individually, 50 mM Tris-HCl (pH 8.0), 40 mM KCl, 10 mM MgCl_2_, 0.1 mg/ml BSA, using undamaged or damaged templates. The assay was carried out at 60°C for 10 min or with the time periods indicated for each experiment. The reaction was terminated by addition of 10 μl 2× loading dye solution (1× Tris-borate-EDTA [TBE], 8 M urea, 10 mM EDTA, 0.1% bromophenol blue), followed by denaturation at 95°C for 5 min and immediate chilling on ice. Replication products were resolved by 18% urea polyacrylamide gel electrophoresis and visualized by an Amersham ImageQuant 800 biomolecular imager (Cytiva).

### Proofreading assay.

The proofreading reaction was performed essentially as described for the primer extension assay, except dNTPs were omitted from the reaction mixture.

### Steady-state kinetics analysis.

Steady-state kinetics was performed as described previously (53). To ensure that the reaction was in the linear range, product formation was kept to less than 20% of the starting substrate. For misincorporation and mismatch extension kinetic assay, each 10-μl reaction mixture contained 50 nM 5′ FAM-labeled substrate, 1,000 nM corresponding unlabeled cold DNA substrate with the same DNA sequence, and 350 nM Dpo2 protein. The reaction was initiated by addition of dNTP at various concentrations and was terminated by mixing with 10 μl 2× loading dye solution (1× TBE, 8 M urea, 10 mM EDTA, and 0.03% bromophenol blue) and heating at 95°C for 5 min. Products were resolved in an 18% urea-PAGE gel and visualized by an Amersham ImageQuant 800 biomolecular imager. The percentage of product formation was quantitated using ImageQuant software, and the velocity of dNTP incorporation was calculated by dividing the yield of products formed by the respective time of the reaction at each concentration of dNTP. The data were fitted into the Michaelis-Menten equation using GraphPad Prism software, from which the apparent *k*_cat_ and *K_m_* values were determined. The misinsertion frequency was expressed as ƒ_inc_= (*k*_cat_/*K_m_*)_incorrect_/(*k*_cat_/*K_m_*)_correct_. The intrinsic efficiency of Dpo2 on mismatch extension was calculated as described previously (46) using the equation ƒ^0^_ext_= (*k*_cat_/*K_m_*)_mismatch_/(*k*_cat_/*K_m_*)_matched_, which measures the relative probability of extending mismatched termini in competition with matched termini at equal DNA concentrations and at low levels of dNTP substrate, and has been widely used to evaluate the mispair extension ability of DNA polymerases (46, 54, 55). The substrates used for misincorporation kinetics included P2-T1, P2-T1-A, P2-T1G-A, and P2-T1C, as indicated in Table S2. The substrates used for mismatch extension kinetics included P3-T1N (N = A, T, G or C), P3T-T1N (N = A, T, G or C), P3G-T1N (N = A, T, G or C), and P3C-T1N (N = A, T, G or C).

### Data availability.

All data required to evaluate the conclusions of this study can be found in either the main text or the supplemental material.
