# Supplementary figures and images for: A Well-Conserved Archaeal B-Family Polymerase Functions as an Extender in Translesion Synthesis
Source: mBio. 2022 Jan 18;13(1):e02659-21. doi: 10.1128/mbio.02659-21 (PMC8764526; doi:10.1128/mbio.02659-21)

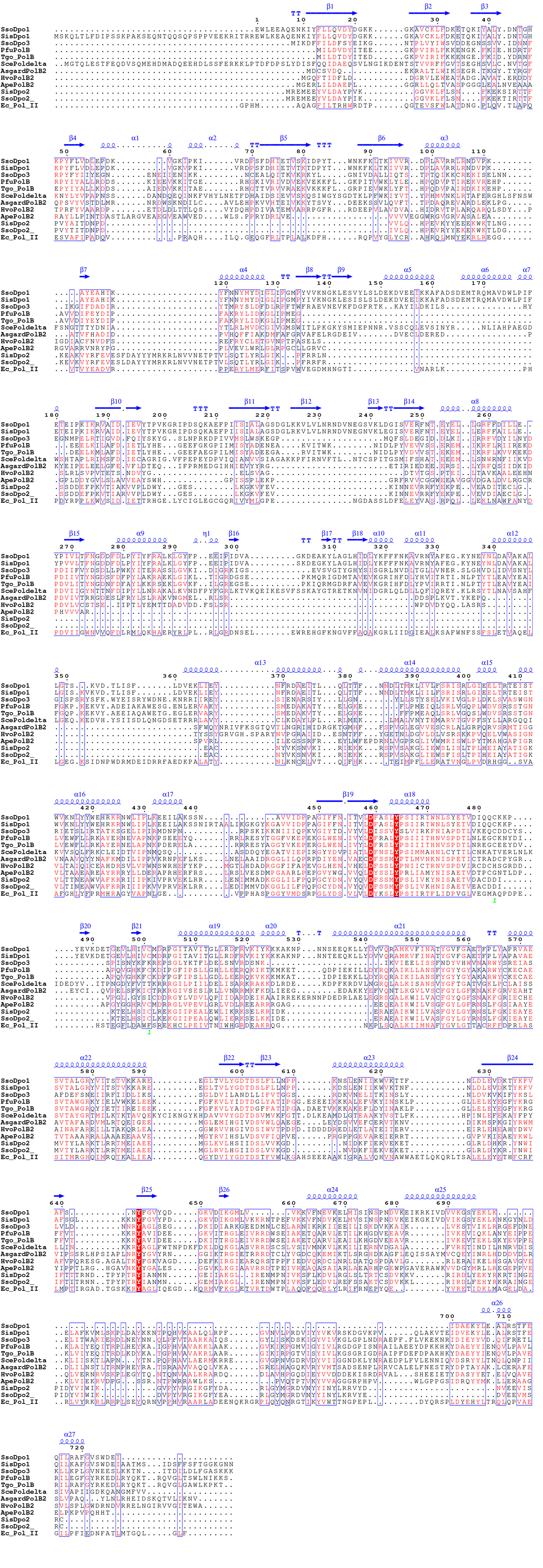

Supplement: FIG S1 [file mbio.02659-21-sf001.tif]

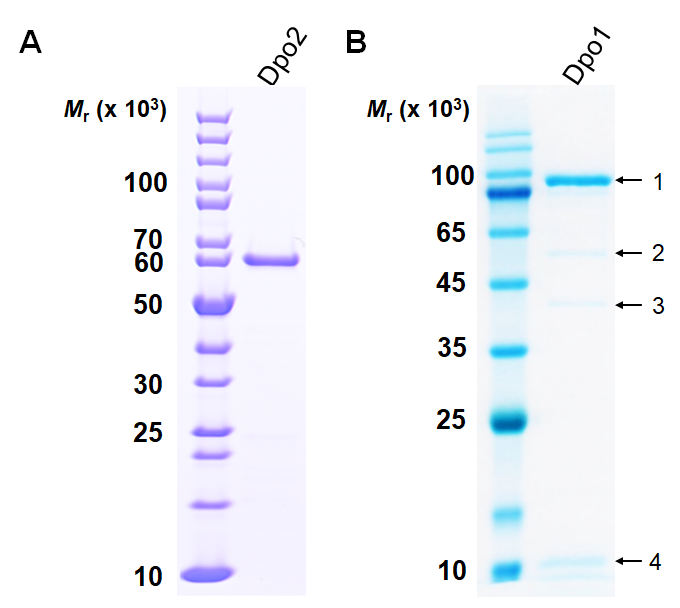

Supplement: FIG S2 [file mbio.02659-21-sf002.tif]

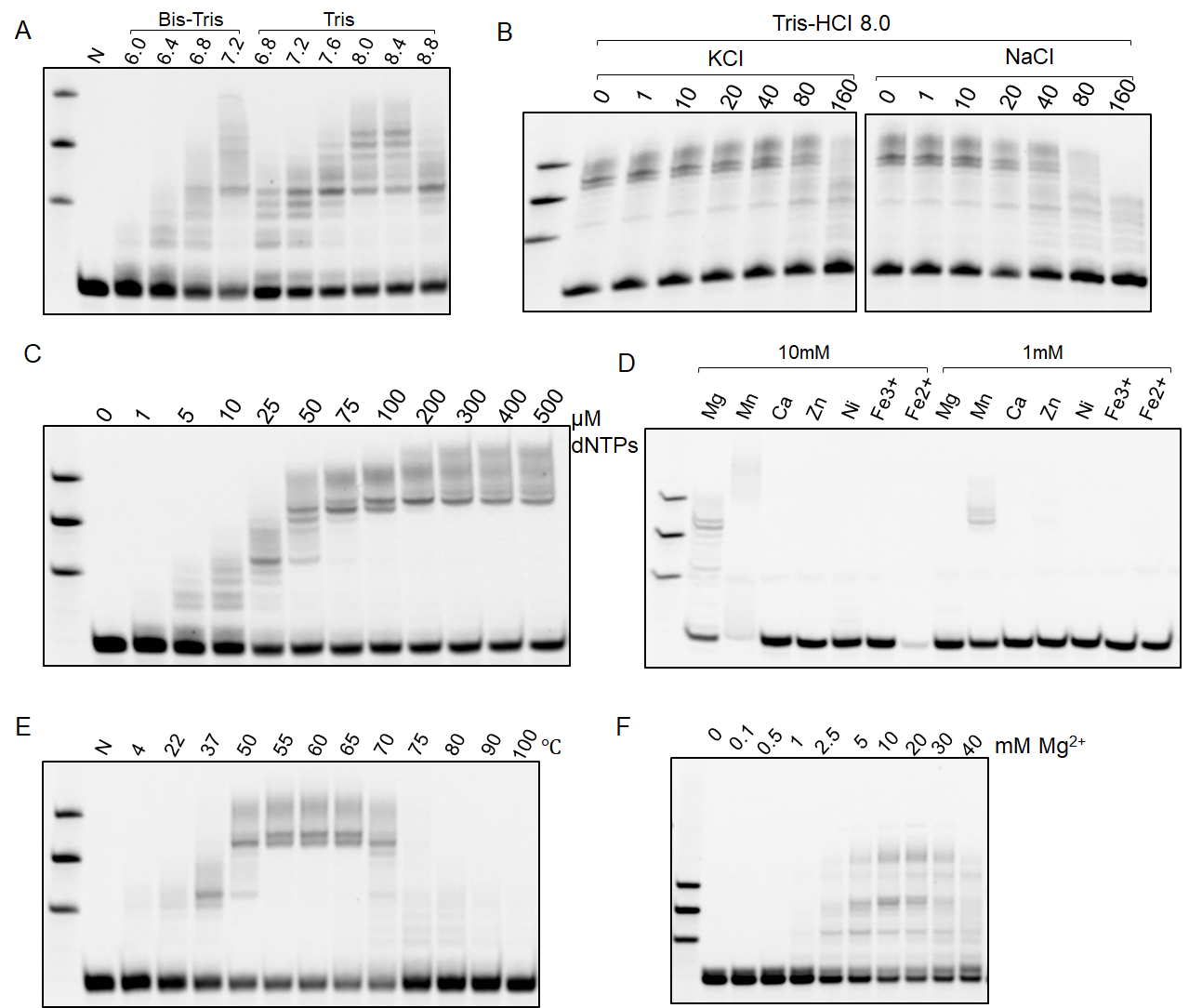

Supplement: FIG S3 [file mbio.02659-21-sf003.tif]

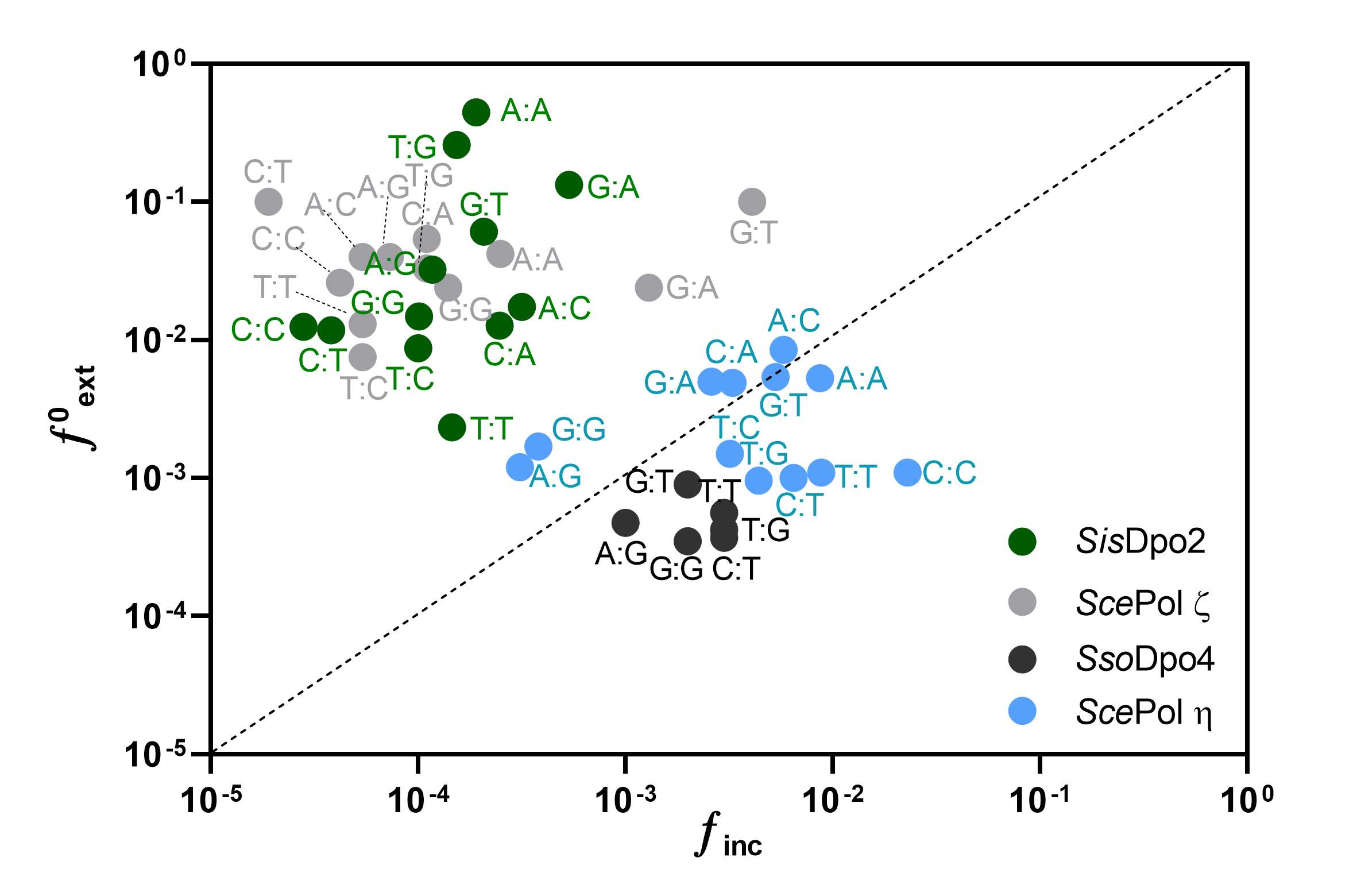

Supplement: FIG S4 [file mbio.02659-21-sf004.tif]

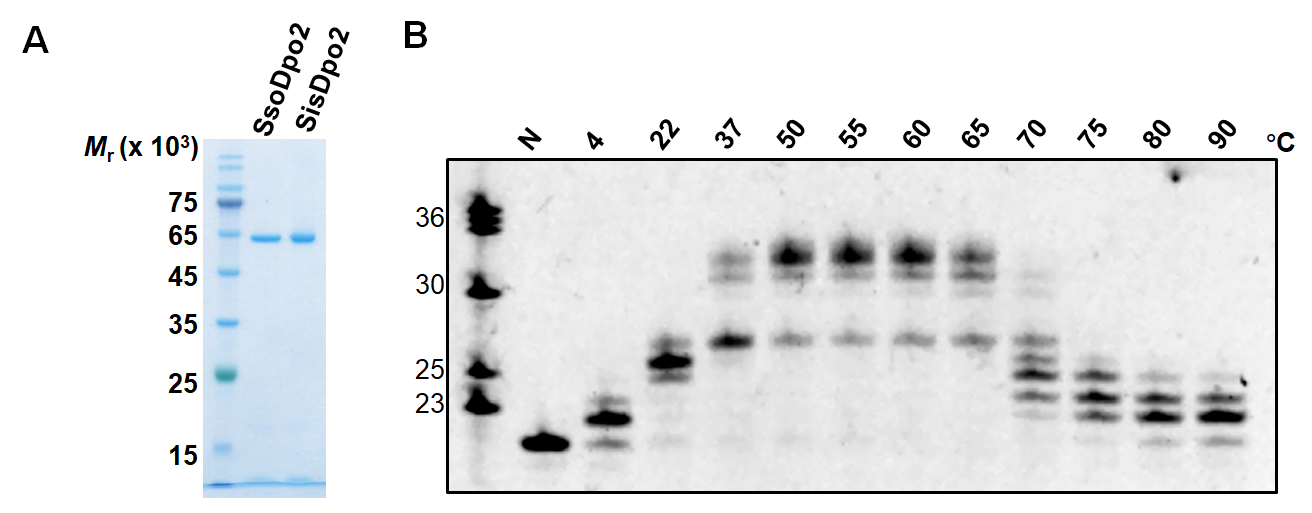

Supplement: FIG S5 [file mbio.02659-21-sf005.tif]

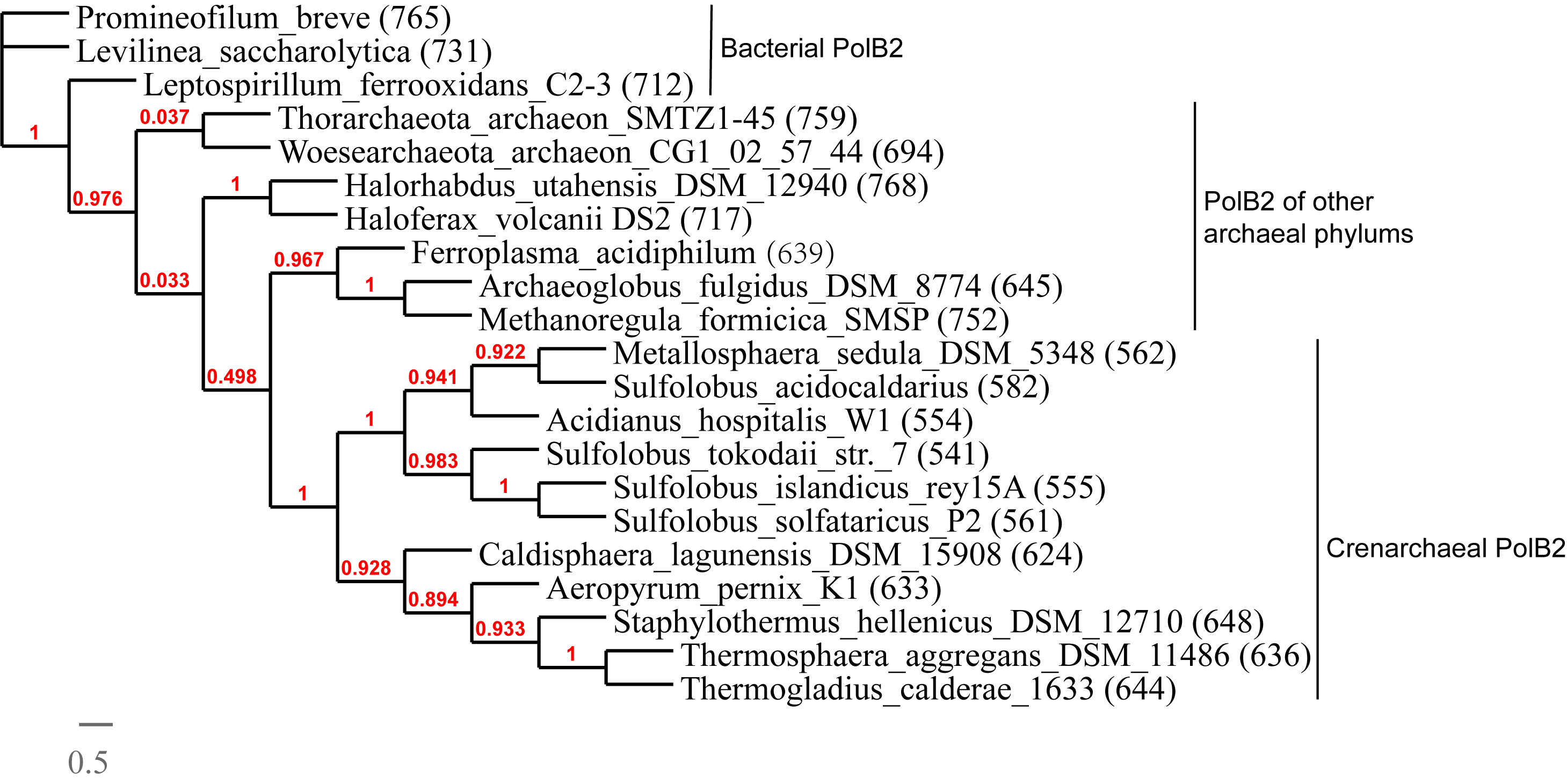

Supplement: FIG S6 [file mbio.02659-21-sf006.tif]
