# Supplementary material for: A Well-Conserved Archaeal B-Family Polymerase Functions as an Extender in Translesion Synthesis
Source: mBio. 2022 Jan 18;13(1):e02659-21. doi: 10.1128/mbio.02659-21 (PMC8764526; doi:10.1128/mbio.02659-21)
Supplement: TABLE S2 [file mbio.02659-21-st002.docx]

**Supplementary Table S2. DNA Substrates Used in This Study**

| **Substrates** | **Sequence (5’ to 3’ for top strand and 3’ to 5’ for bottom strand)** |
| --- | --- |
| P2-T1 | GTGACAGCATCTCATACTCC  CACTGTCGTAGAGTATGAGG**T**CTCCGATTCCACTTT |
| P2-T1A | GTGACAGCATCTCATACTCC  CACTGTCGTAGAGTATGAGG**A**CTCCGATTCCACTTT |
| P2-T1G**^a^** | GTGACAGCATCTCATACTCC  CACTGTCGTAGAGTATGAGG**G**CTCCGATTCCACTTT |
| P2-T1G-A | GTGACAGCATCTCATACTCC  CACTGTCGTAGAGTATGAGG**GA**TCCGATTCCACTTT |
| P2-T1C | GTGACAGCATCTCATACTCC  CACTGTCGTAGAGTATGAGG**C**CTCCGATTCCACTTT |
| P3-T1 | GTGACAGCATCTCATACTCC**A**  CACTGTCGTAGAGTATGAGG**T**CTCCGATTCCACTTT |
| P3-T1A | GTGACAGCATCTCATACTCC**A**  CACTGTCGTAGAGTATGAGG**A**CTCCGATTCCACTTT |
| P3-T1G | GTGACAGCATCTCATACTCC**A**  CACTGTCGTAGAGTATGAGG**G**CTCCGATTCCACTTT |
| P3-T1C | GTGACAGCATCTCATACTCC**A**  CACTGTCGTAGAGTATGAGG**C**CTCCGATTCCACTTT |
| P3T-T1 | GTGACAGCATCTCATACTCC**T**  CACTGTCGTAGAGTATGAGG**T**CTCCGATTCCACTTT |
| P3T-T1A | GTGACAGCATCTCATACTCC**T**  CACTGTCGTAGAGTATGAGG**A**CTCCGATTCCACTTT |
| P3T-T1G | GTGACAGCATCTCATACTCC**T**  CACTGTCGTAGAGTATGAGG**G**CTCCGATTCCACTTT |
| P3T-T1C | GTGACAGCATCTCATACTCC**T**  CACTGTCGTAGAGTATGAGG**C**CTCCGATTCCACTTT |
| P3G-T1 | GTGACAGCATCTCATACTCC**G**  CACTGTCGTAGAGTATGAGG**T**CTCCGATTCCACTTT |
| P3G-T1A | GTGACAGCATCTCATACTCC**G**  CACTGTCGTAGAGTATGAGG**A**CTCCGATTCCACTTT |
| P3G-T1G | GTGACAGCATCTCATACTCC**G**  CACTGTCGTAGAGTATGAGG**G**CTCCGATTCCACTTT |
| P3G-T1C | GTGACAGCATCTCATACTCC**G**  CACTGTCGTAGAGTATGAGG**C**CTCCGATTCCACTTT |
| P3C-T1 | GTGACAGCATCTCATACTCC**C**  CACTGTCGTAGAGTATGAGG**T**CTCCGATTCCACTTT |
| P3C-T1A | GTGACAGCATCTCATACTCC**C**  CACTGTCGTAGAGTATGAGG**A**CTCCGATTCCACTTT |
| P3C-T1G | GTGACAGCATCTCATACTCC**C**  CACTGTCGTAGAGTATGAGG**G**CTCCGATTCCACTTT |
| P3C-T1C | GTGACAGCATCTCATACTCC**C**  CACTGTCGTAGAGTATGAGG**C**CTCCGATTCCACTTT |
| P2CPD-T2CPD (TLS insertion) | GTGACAGCATCTCATACTC  CACTGTCGTAGAGTATGAGTTCTCCGATTCCACTTT |
| P4CPD-T2CPD (TLS extension) | GTGACAGCATCTCATACTCAA  CACTGTCGTAGAGTATGAGTTCTCCGATTCCACTTT |
| P1-T1-oxoG (TLS insertion) | GTGACAGCATCTCATACTC CACTGTCGTAGAGTATGAGoxoGTCTCCGATTCCACTTT |
| P2-T1-oxoG (TLS extension) | GTGACAGCATCTCATACTCC CACTGTCGTAGAGTATGAGoxoGTCTCCGATTCCACTTT |
| P2-T1AP (TLS insertion) | GTGACAGCATCTCATACTCC CACTGTCGTAGAGTATGAGGX**T**CTCCGATTCCACTTT |
| P3-T1AP (TLS extension) | GTGACAGCATCTCATACTCC**A** CACTGTCGTAGAGTATGAGGX**T**CTCCGATTCCACTTT |

Each substrate is annotated with names of its primer and template and the assay in which the substrate is to be used is given in the parentheses. a. For dG incorporation on P2-T1G, there is a propensity for strand slippage mechanism to occur, leading to the pairing of the incoming nucleotide (dG) and the 5’ flanking template base (dC), which is a correct base pairing instead of a mismatched base pair. To avoid that, the P2-T1G-A substrate, in which the 5’ dG was changed to dA, was used instead. TT represents a CPD dimmer; X denotes a synthetic abasic site whereas oxoG indicates an 8-oxodG site.
