# Supplementary material for: A Well-Conserved Archaeal B-Family Polymerase Functions as an Extender in Translesion Synthesis
Source: mBio. 2022 Jan 18;13(1):e02659-21. doi: 10.1128/mbio.02659-21 (PMC8764526; doi:10.1128/mbio.02659-21)
Supplement: TABLE S1 [file mbio.02659-21-st001.docx]

**Supplementary Table S1. Oligonucleotides Used in This Study**

| **Primer strand** | **Sequence (5’ to 3’)** | **Source** |
| --- | --- | --- |
| P1 | 5’ FAM-GTGACAGCATCTCATACTC | Genewiz |
| P2 | 5’ FAM-GTGACAGCATCTCATACTCC | Genewiz |
| P3 | 5’ FAM-GTGACAGCATCTCATACTCCA | Genewiz |
| P3T | 5’ FAM-GTGACAGCATCTCATACTCCT | Genewiz |
| P3G | 5’ FAM-GTGACAGCATCTCATACTCCG | Genewiz |
| P3C | 5’ FAM-GTGACAGCATCTCATACTCCC | Genewiz |
| P2-CPD | 5’ FAM-GTGACAGCATCTCATACTC | Genewiz |
| P4-CPD | 5’ FAM-GTGACAGCATCTCATACTCAA | Genewiz |
| **Template strand** | **Sequence (3’ to 5’)** |  |
| T1-A | 3’ CACTGTCGTAGAGTATGAGG**A**CTCCGATTCCACTTT | Genewiz |
| T1 | 3’ CACTGTCGTAGAGTATGAGG**T**CTCCGATTCCACTTT | Genewiz |
| T1-G | 3’ CACTGTCGTAGAGTATGAGG**G**CTCCGATTCCACTTT | Genewiz |
| T1-G-A | 3’ CACTGTCGTAGAGTATGAGG**G**ATCCGATTCCACTTT | Genewiz |
| T1-C | 3’ CACTGTCGTAGAGTATGAGG**C**CTCCGATTCCACTTT | Genewiz |
| T1-AP | 3’ CACTGTCGTAGAGTATGAGGX**T**CTCCGATTCCACTTT | Genewiz |
| T2-CPD | 3’ CACTGTCGTAGAGTATGAGTTCTCCGATTCCACTTT | GeneLink |
| T1-oxoG | 3’ CACTGTCGTAGAGTATGAGoxoGTCTCCGATTCCACTTT | Sangon |
| **Cloning primers** | **Sequence (5’ to 3’)** |  |
| Dpo1-F | gaatgaggtgaagctcatatgAGTAAGCAACTTACCTTATTTGATATTCC | Tsingke |
| Dpo1-R | ggccgcttgatcagcgtcgacATTATTCCCTTTTCCTCCTGTGAA | Tsingke |
| SsoDpo2-NdeI-F | GGCCACTcatatgCGAGAAATGGAGGAGTACGTAC | Tsingke |
| SsoDpo2-SalI-R | ATTTgtcgacACACCTAGACATCACCTCTTCC | Tsingke |

TT represents a CPD dimmer; X denotes a synthetic abasic site whereas oxoG indicates an 8-oxodG site.
